# Synovial Sarcoma of the Submaxillary Salivary Gland: A Rare Location and Challenging Diagnosis

**DOI:** 10.7759/cureus.59932

**Published:** 2024-05-08

**Authors:** Nassira Karich, Akkouh Nada, Anass Haloui, Noura Seghrouchni, Amal Bennani

**Affiliations:** 1 Department of Pathology, Mohammed VI University Hospital, Faculty of Medicine and Pharmacy of Oujda, Mohammed First University of Oujda, Oujda, MAR

**Keywords:** cancer, aggressive sarcoma, rare sarcoma, principal salivary gland, synovial sarcoma

## Abstract

Synovial sarcoma is a high-grade sarcoma. The periarticular region, deep soft tissues, and the extremities are where it is most frequently found. The head and neck regions are rarely affected and salivary gland localization is rather rare, especially the submaxillary gland. The process of diagnosis and therapeutic management remains challenging, particularly in cases with uncommon tumor locations where the establishment of a universal therapeutic consensus is complicated. Early diagnosis and a multidisciplinary approach can lead to success without locoregional recurrence or distant metastases.

## Introduction

Synovial sarcoma (SS) is a malignant sarcoma of young adults, rarely involving the head and neck, especially the principal salivary glands. According to Setti et al., soft tissue tumors account for 2-5% of salivary gland tumors [1]. Its diagnosis is purely anatomopathological and constitutes a real challenge, as does its therapeutic management, which remains difficult, especially in the advanced stages with a poor prognosis [2]. The aim of our case report is to provide an update on SS, a rarely described sarcoma, in a very rare location: the submaxillary salivary gland.

## Case presentation

A 24-year-old female presented with a painless, left mandibular swelling. Her past history involved recurrent otitis since 2019, treated with local and general antibiotics. The swelling was present for five months prior to her presentation, marked by a rapid increase in size, with no dyspnea, dysphagia, or alteration in general condition. On clinical examination, the mass appeared deep, with no inflammatory signs on the adjacent skin and no local or distant adenopathy.

**Figure 1:**
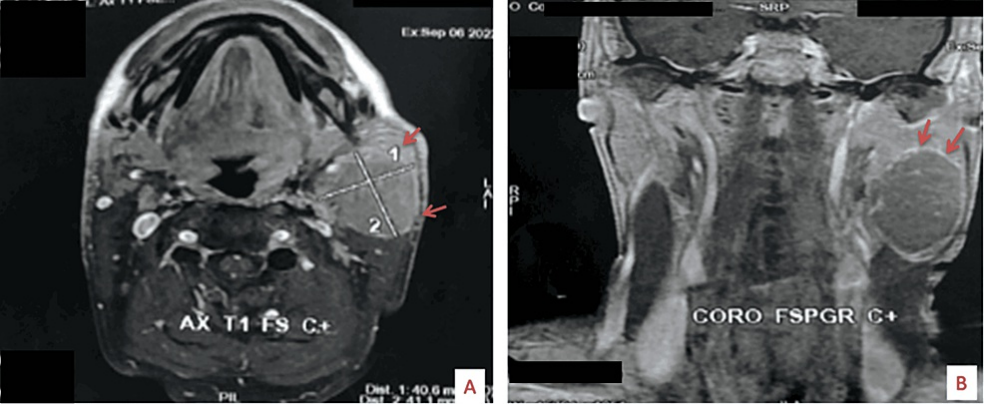
MRI coronal (A) and axial (B) sections showing a 6 cm synovial sarcoma of the left submaxillary gland (red arrows).

On magnetic resonance imaging (MRI), the tumoral process was observed at the expense of the left submaxillary gland, not well circumscribed in places, and had a heterogeneous appearance (Figure 1). It measured 60x50x44 mm on the long axis, with central and peripheral necrotic areas occupying less than 10% of the lesion volume. It was hypervascular, hypointense in T1 and T2, and hyperintense in diffusion with ADC (apparent diffusion coefficient) restriction. The mass was in contact with the muscles of the left floor of the mouth, exerting a mass effect on the left lateral wall of the oropharynx. Brain, neck, and chest-abdomen-pelvis CTs were performed for evaluation of extension and metastasis was not seen.

A biopsy of the mass was performed with an extemporaneous examination, which revealed a very monotonous, highly cellular tumor proliferation of fibroblastic cells with enlarged and irregular nuclei. Given this appearance, the tumor mass was completely resected.

**Figure 2:**
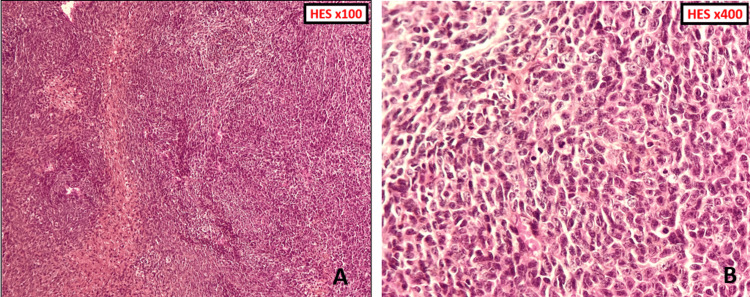
(A) Microphotography showing the hypercellular tumor proliferation organized in intersecting fascicles; (B) At high power, tumor cells are ovoid to fusiform, moderately atypical with mitotic figures.

Macroscopic examination revealed salivary parenchyma infiltrated by a whitish multinodular lesion of hard consistency. Histological study revealed principal salivary parenchyma extensively infiltrated by highly cellular tumor proliferation organized in intersecting fascicles (Figure 2A). Tumor cells were generally monomorphic, ovoid to fusiform, mildly atypical, with nuclei that were sometimes hyperchromatic, sometimes with vesicular chromatin, and fine nucleoli. The cytoplasm was sometimes sparse and eosinophilic, sometimes clear. The mitotic score was estimated at 9 mitoses/mm²: Score 1 according to the Fédération Nationale des Centres de Lutte Contre le Cancer (FNCLCC) (Figure 2B).

**Figure 3:**
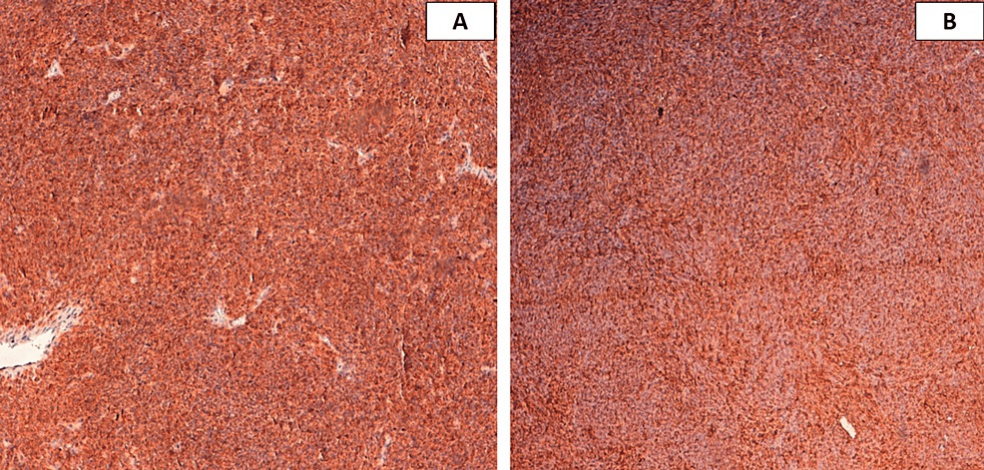
Tumor cells strongly and diffusely expressing BCL2 (A) and CD99 (B) (Brown staining).

**Figure 4:**
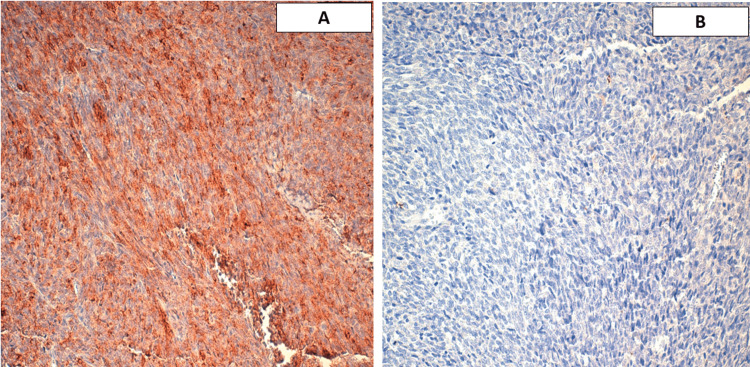
Tumor cells expressing EMA heterogeneously (brown color) (A) and not expressing pan-cytokeratin (no brown color) (B). EMA: epithelial membrane antigen

A few thin-walled vessels were present within the tumor proliferation, with hemangiopericytic appearance, with fibrous and oedematous foci. No tumor necrosis or epithelial component was found, despite good sampling of the specimen. An immunohistochemical study was done, showing positive staining of tumor cells by BCL2 (B-cell lymphoma 2), CD99 (Figure 3), and focally by smooth muscle actin (SMA), and epithelial membrane antigen (EMA) (aka MUC1) (Figure 4). Tumor cells were negative for pan-cytokeratin, desmin, CD34, PS100 (S100 Ca-binding proteins), and STAT6 (signal transducer and activator of transcription 6).

In the light of these histological and immunohistochemical data, the diagnosis of an SS of Grade 2 (3+1+0) according to the FNCLCC, was retained. Testing for the translocation t(X;18) (p11.2; q11) was not performed.

After a discussion of the case in a multidisciplinary consultation meeting, the therapeutic decision was adjuvant radiotherapy. The patient was referred for a pre-radiotherapy consultation and a prescription for dental treatments was requested prior to the beginning of treatment. The patient was subsequently lost to follow-up. She returned for an otorhinolaryngology consultation for a recurrence of a similar mass at the same site. This mass was resected, and the anatomopathological examination of the specimen was the same as the initial result. The patient is now undergoing radiotherapy.

## Discussion

Tumors of the principal salivary glands account for approximately 4% of all cervicofacial tumors. Of these, the submaxillary salivary gland is implicated in just 22% of cases. In 80% of cases, the tumors are benign, and in 95% of cases, they are of epithelial origin [1]. Just 1% of cervicofacial malignant tumors are sarcomas [3]. In the salivary glands, they include, in order of frequency, hemangiopericytoma, malignant schwannoma, malignant fibrous histiocytoma, rhabdomyosarcoma, angiosarcoma, and lastly, SS [1].

SS is a high-grade histological variety of sarcoma, accounting for 10% of all soft tissue sarcomas (all sites included) and less than 0.1% of cervicofacial cancers. In 70% of cases, it occurs in deep soft tissues and in the extremities, essentially in the periarticular area. It is located in the trunk in 15% of cases and in the head and neck in 7%; in only a very few cases, it is found in the submaxillary salivary region [4].

There have been reports in the literature of other unusual localizations, cervicofacial and elsewhere, such as the buccal mucosa [5], the maxillary sinus [6], the mandible [7], the tongue [8], and the floor of the mouth [9]. In addition, it also affects the small bowel and stomach, kidney, adrenal gland, internal and external genital organs, lung, bone, and heart, as well as the central nervous system and peripheral nerves [10-12].

SS affects both sexes equally and can occur at any age; in 77% of cases before the age of 50, it predominates in adolescents and young adults and accounts for 1.6% of sarcomas in patients aged over 50 [4]. Although its pathogenesis is unclear, the potential hypothesis that it arises from the malignant degeneration of a multipotent mesenchymal cell may explain its incidence outside of the regions normally occupied by synovial tissue [13].

Clinically, it usually presents as a slowly growing mass, particularly in its early stages, that might or might not be followed by an aggressive growing phase. Compared to the extremities and joints, the cervicofacial region has a more rapid and aggressive pattern of progress. It may or may not be painful [14]. It may differ in size from less than 1 cm to more than 10 cm, and it is usually well-limited [15]. Because of its small size, the tumor can be misdiagnosed, delaying diagnosis and negatively affecting the prognosis [16].

Macroscopically, the tumor may be of different colors: beige, yellowish, pinkish, or greyish. It may be nodular, multinodular, or multicystic. It may contain calcifications, fibrous, myxoid, or hemorrhagic changes [17].

According to the WHO [18], there are two subtypes: Monophasic SSs, which are the most prevalent and pose the most diagnostic difficulties, and Biphasic SSs which account for about 20-30% of cases. Biphasic SSs present variable proportions of epithelial and sarcomatous components.

The epithelial component is arranged in nests and/or cords and/or glands with rare papillary structures. The epithelial cells are either cubic or columnar, with round to ovoid nuclei, vesicular chromatin, and eosinophilic cytoplasm that are more abundant than those of the sarcomatous component. In rare cases, squamous metaplasia is seen in epithelial cells [18].

The sarcomatous component (with the same characteristics in monophasic and biphasic SSs) is hypocellular and/or hypercellular, arranged in long, intersecting fascicles, sometimes with a palisading arrangement of cells. The cells are monomorphic, having elongated hyperchromatic nuclei, with fine nucleoli and uniformly dispersed granular chromatin, along with scant cytoplasm. Collagenous, hemorrhagic, and myxoid changes vary from one tumor to another, as does the presence of mast cells, calcifications, osteoid bone (which may lead to a wrong diagnosis of osteosarcoma [19]), or staghorn vessels (which may be confused with a solitary fibrous tumor), and this was the reason why we thought, in our patient’s case, to eliminate a solitary fibrous tumor by STAT6.

It is possible to observe poorly differentiated areas with notable hypercellularity, marked cytonuclear atypia, more than 6 mitotic figures/mm², as well as tumor necrosis foci [20]. These patches might be composed of epithelioid cells, small round hyperchromatic cells (similar to Ewing’s sarcoma), or fascicular spindle cells (similar to malignant peripheral nerve sheath tumors) [21].

The vast majority of SSs are positive for TLE1 (transducin-like enhancer of split 1) with nuclear staining, BCL2, and CD99 which may show membrane staining as in Ewing’s sarcoma. However, these markers are not specific [22]. SS expresses EMA more than pan-cytokeratin, which is positive in 50% of cases. Focal expression of PS100 is seen in 40% of cases, SMA in less than 50%, desmin is rarely positive, and H-Caldesmon is always negative. CD34 is rarely positive in monophasic SSs [22]. On the cytogenetic side, SS is characterized in 95% of cases by a specific chromosomal translocation t(X;18) (p11.2; q11.2). Molecularly, it is characterized by a specific SS18-SSX1/2/4 fusion gene [23]. However, if the diagnosis is confirmed by clinical, histological, and/or immunohistochemical findings, molecular testing is not required [2], as in the case of our patient.

After the initial diagnosis, recurrences and metastases (on lung, lymph nodes, or bones) may appear a few months to several years (more than 10 years) later. The key determinants of prognosis are localization (more aggressive and therefore a more guarded prognosis in the cervicofacial region [24]) and tumor size (a good prognosis at <5cm in long axis) at the time of diagnosis, in addition to the patient’s age and histological subtype, and undoubtedly the grade of the tumor according to the FNCLCC [20].

Tumors with more than 20% of poorly differentiated areas are more aggressive. The best therapeutic results are obtained with tumors that present < 6 mitoses/mm² without a tumor necrosis zone [20].

Early diagnosis followed by a multidisciplinary approach to establish an appropriate treatment (surgery/ surgery+adjuvant radiotherapy/surgery+chemotherapy) can give hope of a cure, given that many studies have analyzed these different therapeutic modalities and have not been able to conclude that one strategy is superior to another [25]. On the other hand, surgical resection is followed by local recurrence in an average of 75% of cases within two years, which is the reason why Daveau et al., in their case report, support the strategy of adjuvant radiotherapy which reduces recurrence and improves overall survival [26].

With regard to chemotherapy, a meta-analysis of all sarcomas, 10% of which were SSs (all localizations considered), showed that doxorubicin significantly delayed loco-regional recurrence and metastases [27]. Like with all exceptions, cervicofacial localization, and more so, salivary localization, remains poorly mentioned in the literature, and the establishment of a universal therapeutic consensus is still very challenging and complicated, particularly when it comes to chemotherapy [24]. Globally, SSs of the head and neck are associated with a 40-60% five-year survival rate [28-32].

## Conclusions

SS is a rare sarcoma and can be seen in sites other than articular and periarticular localizations. The diagnosis is still challenging, particularly in its monophasic variant and even more so in poorly differentiated forms. Additionally, unusual localization increases the difficulty of diagnosis. Its prognosis varies according to several parameters, and generally, the cervicofacial localization has a worse prognosis than the other usual localizations.
